# Supplementary material for: Deletion of wfs1 Impairs Oligodendrocyte Precursor Cells Dorsal Distribution and Myelination Through the wfs1-hmgcs1 Axis in Zebrafish
Source: Biology (Basel). 2026 Aug 16;15(16):1402. doi: 10.3390/biology15161402 (PMC13510151; doi:10.3390/biology15161402)
Supplement: Supplementary file 1 [file biology-15-01402-s001.zip › File S1-Transcriptome sequencing data and differentially expressed genes (DEGs) in wfs1-- zebrafish at 5 dpf/2.KEGG/wfs1KOvsWT/ALL/src/dre00270.html]

dre00270


- dre:561410

- Up regulated genes

561410\_1(1.20432290851387)

- dre:445818

- Up regulated genes

445818(1.93771996696489)

- dre:550524

- Down regulated genes

550524\_1(-2.57926437647488)

- dre:103911233

- Up regulated genes

103911233(1.84859647244981)
- dre:571201

- Up regulated genes

103911241(5.46067952567297)

- Down regulated genes

571201(-9.03201015725177)

- dre:334195

- Up regulated genes

334195\_1(2.11046533957304)

- Down regulated genes

334195(-1.02556051088404)
- dre:678634

- Down regulated genes

678634(-2.56566805889366)

- dre:791167

- Down regulated genes

791167(-8.30993863819111)

- dre:317744

- Up regulated genes

317744\_1(3.31151182596513)
- dre:30659

- Up regulated genes

30659\_1(3.25829209683907)

- dre:431773

- Up regulated genes

431773\_1(1.99676342400628)

- Down regulated genes

431773(-4.19518690776961)

- dre:563642

- Up regulated genes

563642(1.74231278020234)

- dre:406688

- Down regulated genes

406688(-1.12341806422973)

- dre:445818

- Up regulated genes

445818(1.93771996696489)

- dre:406688

- Down regulated genes

406688(-1.12341806422973)

- dre:103911233

- Up regulated genes

103911233(1.84859647244981)
- dre:571201

- Up regulated genes

103911241(5.46067952567297)

- Down regulated genes

571201(-9.03201015725177)

- dre:445818

- Up regulated genes

445818(1.93771996696489)

- dre:406688

- Down regulated genes

406688(-1.12341806422973)

- dre:335715

- Up regulated genes

335715(3.34138689148967)

- dre:450084

- Up regulated genes

450084\_1(4.00880053586154)

- dre:103911233

- Up regulated genes

103911233(1.84859647244981)
- dre:571201

- Up regulated genes

103911241(5.46067952567297)

- Down regulated genes

571201(-9.03201015725177)

Close
